# *Zr*Fsy1, a High-Affinity Fructose/H^+^ Symporter from Fructophilic Yeast *Zygosaccharomyces rouxii*

**DOI:** 10.1371/journal.pone.0068165

**Published:** 2013-07-02

**Authors:** Maria José Leandro, Hana Sychrová, Catarina Prista, Maria C. Loureiro-Dias

**Affiliations:** 1 CBAA, Instituto Superior de Agronomia, Universidade Técnica de Lisboa, Lisbon, Portugal; 2 Department of Membrane Transport, Institute of Physiology AS CR, v.v.i., Prague, Czech Republic; University of Florida, United States of America

## Abstract

*Zygosaccharomyces rouxii* is a fructophilic yeast than can grow at very high sugar concentrations. We have identified an ORF encoding a putative fructose/H^+^ symporter in the *Z. rouxii* CBS 732 genome database. Heterologous expression of this ORF in a *S. cerevisiae* strain lacking its own hexose transporters (*hxt*-null) and subsequent kinetic characterization of its sugar transport activity showed it is a high-affinity low-capacity fructose/H^+^ symporter, with K_m_ 0.45±0.07 mM and V_max_ 0.57±0.02 mmol h^−1^ (gdw) ^−1^. We named it *Zr*Fsy1. This protein also weakly transports xylitol and sorbose, but not glucose or other hexoses. The expression of *ZrFSY1* in *Z. rouxii* is higher when the cells are cultivated at extremely low fructose concentrations (<0.2%) and on non-fermentable carbon sources such as mannitol and xylitol, where the cells have a prolonged lag phase, longer duplication times and change their microscopic morphology. A clear phenotype was determined for the first time for the deletion of a fructose/H^+^ symporter in the genome where it occurs naturally. The effect of the deletion of *ZrFSY1* in *Z. rouxii* cells is only evident when the cells are cultivated at very low fructose concentrations, when the *Zr*Fsy1 fructose symporter is the main active fructose transporter system.

## Introduction

*Zygosaccharomyces rouxii* and *Z. bailii* are food spoilage yeasts that can grow under harsh conditions that are restrictive for most yeast species due to their extreme osmotolerance and resistance to weak-acid preservatives [1]. *Z. bailii* and *Z. rouxii* strains were isolated from high-sugar environments, 72 and 90% (w/v) glucose, respectively. These yeasts are fructophilic, *i.e.* they consume fructose faster than glucose, whereas the main fermentative yeast *Saccharomyces cerevisiae* exhibits glucophilic behavior [2]. The fructophily of these *Zygosaccharomyces* is based on the kinetics and substrate specificity of their sugar transporters, mainly those mediating the uptake of sugars via facilitated diffusion (facilitators). In *Zygosaccharomyces,* fructose uptake is mediated by high-capacity low-affinity fructose-specific facilitators, whereas glucose is transported via low-capacity high-affinity non-specific sugar facilitators, which also accept fructose and 2-deoxyglucose as their substrates [3] (S. Sousa-Dias and M. C. Loureiro-Dias, unpublished data).

Most of the sugar transporters characterized so far belong to the Sugar Porter (SP) family, the largest member of the Major Facilitator Superfamily [4]. They are usually single integral membrane proteins with two sets of six hydrophobic transmembrane-spanning α-helices and five sequence-conserved motifs (RXGRR between transmembrane regions two and three; PESPRXL at the end of transmembrane region six; PETKGXXXE at the end of transmembrane region twelve; R-X_3_-G-X_3_-G-X_6_-P-X-Y-X_2_-E-X_6_-R-G-X_6_-Q-X_5_-G through transmembrane domains four and five; [LI]-Q-X_2_-Q-Q-X-[ST]-[GN]-X_3_-Y-Y-F in transmembrane region seven). Members of the SP family operate via active proton symport or energy-independent facilitated diffusion mechanisms [5].

In general sugar-proton symporters, which are able to transport the sugar against its concentration gradient simultaneously with the movement of (a) proton(s), only operate when relatively low concentrations of sugar are available [5]. In contrast, facilitators are employed when a sufficient amount of sugars is present, so the transported molecules are rapidly metabolized inside the cells and thus the inward gradient of sugar necessary for an efficient facilitated diffusion is maintained.

Fructose/H^+^ symporters, which are able to discriminate between fructose and other hexoses, have been identified and partly characterized in several yeast species. The first was Fsy1 (fructose symport) from *Saccharomyces pastorianus* PYCC 4457, the type strain of *S. carlsbergensis*. The corresponding gene was isolated by functional complementation of a hexose-transporter-less *S. cerevisiae* strain. *Sp*Fsy1 shares a low sequence similarity to *S. cerevisiae* Hxt proteins [6] and it mediates a high-affinity D-fructose uptake, also accepting sorbose as substrate. Its expression only occurs at low sugar concentrations [7]. *Saccharomyces bayanus* PYCC 4565 (a hybrid containing the genomic DNA of at least two related species, *S. eubayanus* and *S. uvarum*) has an *FSY1* allele identical to that of *S. pastorianus* PYCC 4457 and is also present in wild-type *S. eubayanus*, whereas the *FSY1* allele of *S. uvarum* CBS 7001 is slightly different (91% identity). All the encoded transporters from the four *Saccharomyces* species are biochemically indistinguishable. Fsy1 proteins favor a stoichiometry of 1∶1, they are used when fructose is scarce, and they have a scavenging role in conditions where the glycolytic flux is low and the metabolism is respiratory [8]. Fsy1 also has a close relative in *Kluyveromyces lactis* encoded by the *FRT1* gene, which is induced by glucose, fructose and, to a lesser extent, by galactose [9].

The *FSY1*-type of gene is present in the genomes of other yeasts such as Lanchancea (Saccharomyces) waaltii, Scheffersomyces stipitis (Pichia stipitis), Debaryomyces hansenii, Candida albicans and Clavispora lusitanea, of various ascomycetous filamentous fungi, and of the gray mold fungus Botrytis cinerea [5], [10], [11]. The *FSY1* gene therefore seems to appear early in the evolution of Ascomycetes [5]. However, it was not found in the genomes of laboratory S. cerevisiae strains, but only in wine strains such as strain EC1118 [12]. The corresponding gene is repressed by high concentrations of glucose or fructose and induced by ethanol as the sole carbon source. In the S. cerevisiae wine strains, the gene was probably acquired by a horizontal gene transfer and could confer an advantage during the wine fermentation process, when yeasts have to ferment fructose (a non-preferred sugar) in the presence of large amounts of ethanol or under oxidative conditions [13].

Three peculiar sugar transporters, designated Ffz (fructose facilitator of *Zygosaccharomyces*) and suggesting the emergence of a new family of sugar transporters, have been characterized in *Z. rouxii* and *Z. bailii* [14], [15]. In *Z. rouxii,* there are two similar low-affinity high-capacity facilitators: *Zr*Ffz1, which is specific for fructose; and *Zr*Ffz2, which transports both fructose and glucose. These three proteins are phylogenetically unrelated to the Sugar Porter family, and according to their sequence, they are phylogenetically closer to the Drug/H^+^ Antiporter family [14].

The *Z. rouxii* CBS 732 genome, sequenced by the Génolevures consortium, contains other putative hexose transporters that have not yet been characterized, including an ORF that encodes a protein with a high similarity to the *S. pastorianus* Fsy1 fructose/H^+^ symporter. In this paper we describe the cloning and characterization of the fructose proton symporter *Zr*Fsy1 from Z. *rouxii* CBS 732 and analyse its expression and the phenotypes of its deletion in this fructophilic yeast. To our knowledge, we describe for the first time a clear phenotype determined for the deletion of a fructose/H^+^ symporter in the genome where it occurs naturally.

## Materials and Methods

### Strains and Growth Media

*Z. rouxii* CBS 732^T^ was used for the isolation of the gene encoding the putative fructose/H^+^ symporter. The *S. cerevisiae* BW31a strain (*MATa leu2-3/122 ura3-1 trp1-1* his3-11/15 *ade2-1 can1-100 GAL SUC2 mal10 ena1-4Δ*::*HIS3 nha1*::*LEU2*; [16]) was used for the construction of plasmids by homologous recombination. The *S. cerevisiae hxt*-null EBY.VW4000 (CEN.PK2-1C *hxt17Δ hxt13Δ hxt15Δ hxt16Δ hxt14Δ hxt12Δ hxt9Δ hxt11Δ hxt10Δ hxt8Δ hxt514Δ hxt2Δ hxt367Δ gal2Δ slt1Δ agt1Δ ydl247wΔ yjr160cΔ*; [17]) strain was used to express the putative fructose transporter.

Yeasts strains were grown in minimal YNB (yeast nitrogen base without amino acids containing the indicated carbon source and the required supplements) or in rich YPD (yeast extract, peptone, dextrose) or in YPM (yeast extract, peptone, maltose) media. Sugar concentrations are given as percentages (w/v). *Escherichia coli* XL1-Blue (Stratagene) was used as the host for plasmid amplification. *E. coli* transformants were grown in standard Luria–Bertani medium supplemented with ampicillin (100 µg ml^−1^). The putative fructose transporter was deleted in the *Z. rouxii* UL4 strain [18].

### Growth Assays

Yeast cells were grown at 30°C and their growth was monitored either in drop tests on solid media or in liquid media using a ELx808 Absorbance Microplate Reader (BioTek Instruments, Winooski, VT, USA) as described in [19] or by measuring optical densities at 640 nm in an Ultrospec 2100 pro (Amersham Biosciences) spectrophotometer.

### DNA Manipulations

DNA manipulations were performed according to standard protocols [20]. Genomic DNA and plasmid DNA from yeast cells were isolated as described in [21]. High-fidelity DNA polymerase Phusion F-530 (Finnzymes) was used to avoid mismatch base pairing during the synthesis of PCR products. Plasmid DNA from *E. coli* was isolated using a GenElute™ Plasmid Miniprep Kit (Sigma-Aldrich).

### Plasmid and Strain Construction

The plasmids used for cloning were pGRU1 and pNHA1-985GFP (derived from the pGRU1 plasmid, harboring C-terminal GFP-tagged *ScNHA1* driven by its own promoter; [22]). Plasmids containing the ZYRO0C00374*g* (*ZrFSY1*) gene were constructed by homologous recombination in *S. cerevisiae* BW31a [23]. *ZrFSY1* (amplified by PCR with primers ZrFSY1-N-F and ZrFSY1-R) was inserted behind the *ScNHA1* promoter into the pNHA1-985GFP plasmid (previously digested with *Pvu*II), resulting in pZRS1-N. The same gene (amplified by PCR with primers ZrFSY1-S-F and ZrFSY1-R) with its own promoter (869 bp long) was also inserted into the pGRU1 plasmid (previously digested with *Bam*HI), resulting in the pZRS1-S plasmid. The primers (obtained from Sigma-Aldrich) used for plasmid constructions are listed in Table S1. All constructed plasmids and control plasmids pGRU1 and pNHA1-985GFP were transformed into the *S. cerevisiae hxt*-null EBY.VW4000 strain. *S. cerevisiae* transformations were performed as described in [24].

### Construction of *Z. rouxii ZrFSY1* Deletion Strain S1

*Z. rouxii* was transformed by electroporation as described in [24]. The deletion of the *Z. rouxii ZrFSY1* gene in the UL4 strain [18] was performed with a PCR-amplified loxP-kanMX-loxP deletion cassette [25]. Primers (obtained from Sigma-Aldrich) used for the cassette amplifications are listed in Table S1 (ZrFSY1-Kan-F and ZrFSY1-Kan-R) and the pUG6 plasmid [26] was used as a template. The pZCRE plasmid expressing the cre recombinase was used to remove the integrated kanMX marker [25], and the replacement of the original gene with the loxP sequence was confirmed by diagnostic PCR with the following combination of primers: ZrFSY1-368-upF/KANX-R1; KANX-F1/ZrFSY1-246d-R; ZrFSY1-368-upF/ZrFSY1-246d-R (Table S1). The *Z. rouxii Zrfsy1ΔΔ::loxP* deletion strain obtained was designated S1.

### Microscopy

For the visualization of GFP-tagged transporters, mid-exponential phase cells were observed with an Olympus AX70 fluorescent microscope, using a U-MWB fluorescence cube with an excitation filter of 450-480 nm and barrier filter at 515 nm.

For the observation of morphology changes, phase contrast images of mid-exponential grown cells were captured using an inverted fluorescence microscope (Axiovert 200; Carl Zeiss, Germany) equipped with a CoolSNAP EZ digital camera (Photometrics, USA). Images were acquired using MetaFluor software (Universal Imaging Corp., Buckinghamshire, UK).

### Sugar Transport Assays

Initial [U-^14^C] fructose (GE Healthcare formerly Amersham Biosciences) uptake rate and inhibition assays were performed as described in [14].

The existence of H^+^ movements associated with initial sugar/polyol uptake was assessed with a pH meter when adding sugar/polyol pulses to unbuffered cell suspensions, as described in [27]. Cells were harvested (10 000 *g*, 5 min, 4°C) at OD_640_ ∼ 0.8, washed twice with ice-cold water, kept on ice for at least one hour and resuspended in water to a final concentration of about 25 mg (dw) ml^−1^. 0.3 ml of cell suspension was mixed with 0.68 ml of demineralized water and pH adjusted to 5 with HCl or NaOH, in a 3 ml capacity water-jacketed chamber maintained at 25°C, with magnetic stirring. After obtaining a stable baseline, 20 µl of a 500 mM sugar/polyol solution was added (10 mM final concentration), pH data were collected with a pHM82 standard pH meter (Radiometer, Copenhagen) and recorded with a potentiometer recorder (BBC Goerz Metrawatt SE 460). Calibration was performed with an HCl solution. Kinetic parameters were estimated using GraphPad Prism version 5.00 (Graphpad Software, www.graphpad.com) for Michaelis-Menten regression analysis.

Dry weight was determined (in triplicate) by placing 100 µl of cell suspensions into pre-weighed aluminium foil cups and drying in a 70°C oven for 24 h.

### Quantification of *ZrFSY1* mRNA by Real-Time PCR

*Z. rouxii* cells were cultivated in YNB medium with 2% glucose until the exponential phase, washed once with sterile cold water, ressuspended in sterile water and used to inoculate new media with various carbon sources (initial OD_640_ 0.02-0.06). Cells in the exponential phase (OD_640_ 0.6–1.2) were frozen in liquid nitrogen. Total RNA was isolated from frozen cells using Trizol Reagent (Invitrogen) and purified with a High Pure RNA Isolation Kit (Roche) according to the manufacturer’s protocol. Purified total RNA was used as template for Real-Time PCR reactions with the *ZrFSY1*-specific primers ZrFSY1-P1 and ZrFSY1-P2 and *ZrACT1*-specific primers ZrACT1-P1 and ZrACT1-P2 (obtained from STAB VIDA, Table S1) using a SensiFAST SYBR & Fluorescein One-Step Kit (BIOLINE), adding 80 ng of purified total RNA per reaction. All reactions were performed in triplicate. The quantitative Real-Time PCR reaction (10 min 45°C (reverse-transcriptase reaction); 95°C 2 min; 40 cycles: 5 s 95°C, 10 s 60°C, 5 s 72°C) was performed in multiplate PCR 96-well clear plates (BIO-RAD) in an iQ™ Multicolor Real-time device (BIO-RAD).

The comparative Ct method was used to quantify gene expression [28]. Gene expression was normalized with respect to the expression of *ZrACT1* (as the reference gene). Normalized expression levels were compared with the lowest level of expression of *ZrFSY1*, which occurred at fructose concentration of 10%.

## Results

### Identification of a Putative *Z. rouxii* Fructose/H^+^ Symporter

In our previous work, we characterized the first two low-affinity fructose transporters from the fructophilic yeast *Z. rouxii* CBS 732: the fructose-specific facilitator *Zr*Ffz1, which is the major system responsible for the high fructose transport capacity of *Z. rouxii;* and *Zr*Ffz2, which facilitates the uptake of both fructose and glucose [14].

When both the *ZrFFZ1* and *ZrFFZ2* genes in *Z. rouxii* are deleted, the obtained strain still grows on glucose and fructose media (results not shown) meaning that other functional hexose transporters are also present in this yeast. We performed a BLASTP search against known sugar transporters belonging to the Sugar Porter family and, besides the four putative hexose transporters similar to *S. cerevisiae* Hxts and the one similar to the hexose sensor *Sc*Snf3, which was reported by Palma *et al.* [29], we found one putative transporter similar to the *Kluyveromyces lactis* glucose/fructose/galactose transporter Hgt1 and another similar to the *S. pastorianus* fructose/H^+^ symporter Fsy1. We decided to clone the identified ORF similar to the fructose/H^+^ symporter *FSY1* and characterize its transport properties.

The ZYRO0C00374*g* ORF (GeneBank accession n° XM_002495633, Gene ID 8202866) had no introns, and due to the high level of similarity between its putative product and *S. pastorianus* Fsy1, we named it *ZrFSY1* (Figure S1). At the protein level, *Zr*Fsy1 is 564 amino acids long, probably contains 12 transmembrane domains (as predicted by the HMMTOP Server v. 2.0, [30]) and shares 75% identity with *S. pastorianus* Fsy1 [6] and fructose symporters from *S. uvarum* and *S. eubayanus*; 73% identity with *S. cerevisiae* Fsy1 EC1118 [13], and 67% identity with the fructose/H^+^ symporter Frt1 from *Kluyveromyces lactis* [9].

### Functional Expression in *S. cerevisiae*

To characterize this putative *Z. rouxii* fructose symporter, we constructed multicopy plasmids harbouring the corresponding ORF (tagged at its 3′prime with the GFP sequence) either driven by the weak and constitutive *ScNHA1* promoter or by the gene's own promoter (869 bp long). The two constructed plasmids, pZRS1-N and pZRS1-S, were used to transform a *hxt*-null *S. cerevisiae* strain (EBY.VW4000 lacking its own hexose transporters and consequently exhibiting drastically reduced growth on media with glucose or fructose as a carbon source [17]). The pNHA1-985GFP plasmid harboring GFP-tagged Sc*NHA1* driven by its own promoter was used as a negative control.

**Figure 1:**
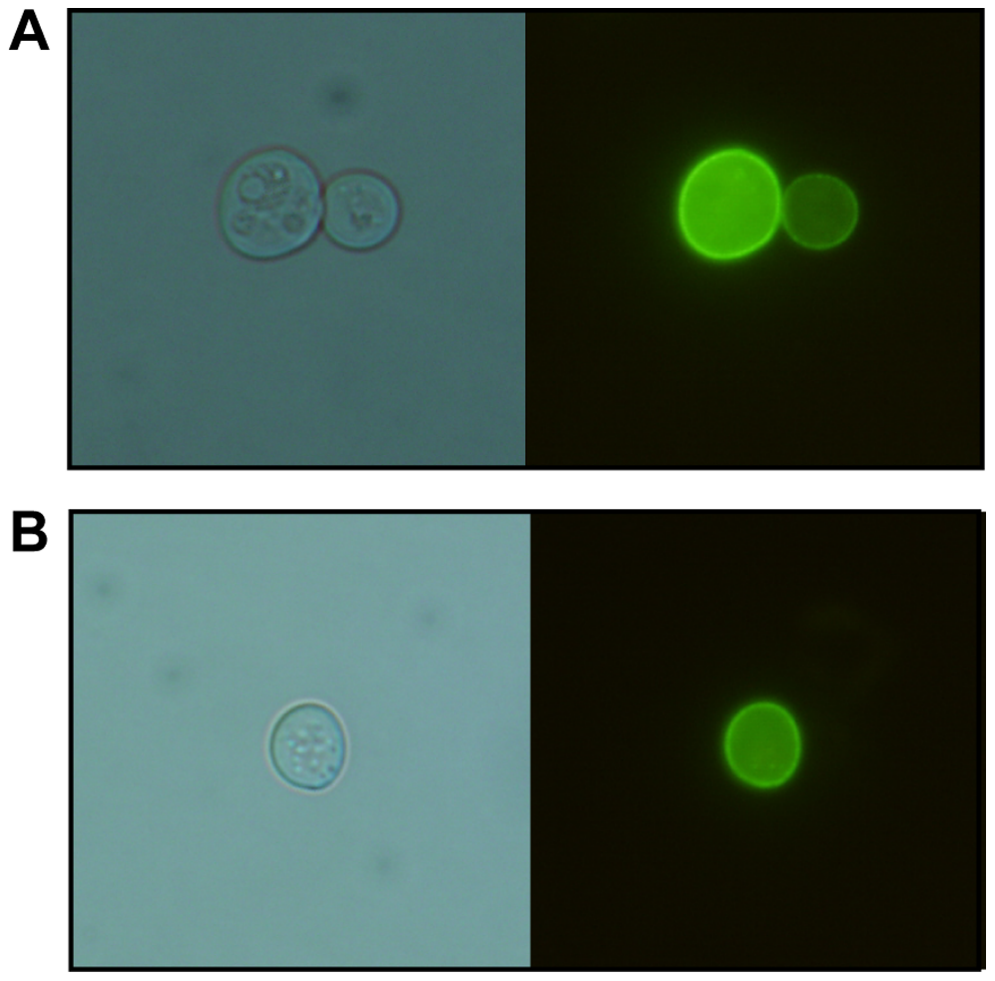
Localization of GFP-tagged *Zr*Fsy1 in *S. cerevisiae* plasma membrane. Phase contrast (left panels) and epifluorescence (right panels) images of *S. cerevisiae hxt-*null strain EBY.VW4000 transformed with multicopy plasmids (A) pZRS1-S (expressing *ZrFSY1* driven by its own promoter) and (B) pZRS1-N (expressing *ZrFSY1* driven by the *NHA1* promoter), grown on YNB medium with 2% maltose.

**Figure 2:**
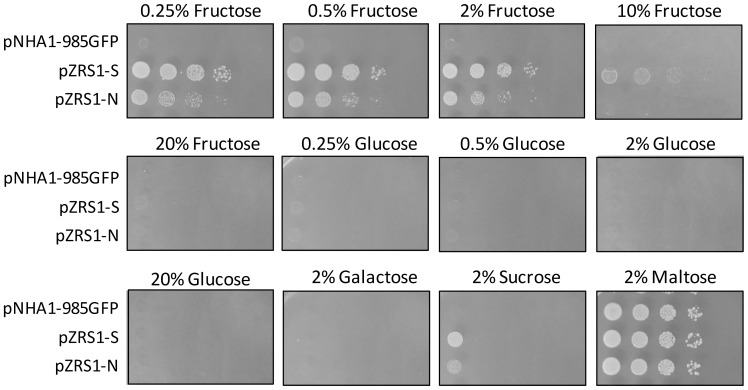
Effect of *ZrFsy1* expression on *S. cerevisiae* growth on solid media. Growth of *S. cerevisiae hxt-*null EBY.VW4000 strain transformed with indicated plasmids grown on YNB media with various carbon sources after 3 days (maltose, fructose and glucose media) or 8 days (sucrose and galactose media) of incubation at 30°C. Results from one of two independent experiments are shown.

GFP-tagging confirmed the predicted plasma-membrane localization of the *Zr*Fsy1 protein, both when expressed from its own promoter or from the *NHA1* promoter (Figure 1).The overexpression of the *Z. rouxii* gene was not toxic to *S. cerevisiae* cells, since the growth of cells transformed with either the pZRS1 plasmids or the control plasmid on 2% maltose was almost the same (Figure 2, last panel).

**Figure 3:**
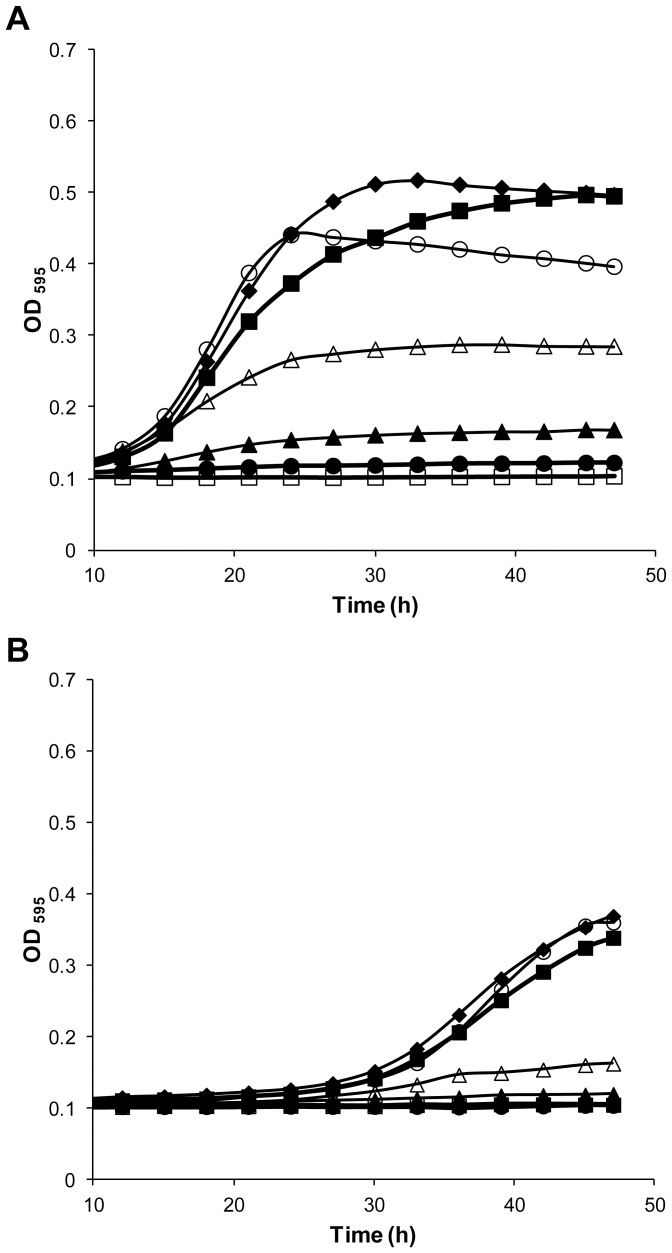
Effect of *ZrFsy1* expression on *S. cerevisiae* growth on liquid media. Growth curves of *S. cerevisiae hxt*-null EBY.VW4000 strain expressing *ZrFSY1* driven by its own promoter (A) or driven from *NHA1* promoter (B) in YNB medium supplemented with 2% glucose (□) or various concentrations of fructose: 0.25% (○), 0.5% (♦), 2% (▪), 5% (△), 10% (▴) and 20% (•). Data are representative of two independent experiments.

Detailed growth assays with various sugars, at several concentrations, on solid (Figure 2) and in liquid media (Figure 3) show that *Zr*Fsy1 expression in a *hxt*-null *S. cerevisiae* strain promoted growth only on fructose, indicating that it is a functional fructose transporter that does not transport glucose or galactose. *S. cerevisiae* EBY.VW4000 cells expressing *ZrFSY*1 also grow slowly on a sucrose medium, as they secrete invertase needed to hydrolyze sucrose to glucose and fructose (Figure 2). When the expression of *ZrFSY1* was controlled by its own promoter (pZRS1-S), the cells were able to grow faster on media with low fructose concentrations than cells harbouring pZRS1-N (Figures 2 and 3), indicating that the *ZrFSY1* promoter is functional in *S. cerevisiae* and is stronger (or effectively upregulated) than the constitutive promoter of *ScNHA1*.

The fact that the expression of *ZrFSY1* allowed better growth at very low fructose concentrations indicates that the corresponding protein should have a high affinity for fructose. On the other hand, very poor growth was observed at high fructose concentrations, even when a constitutive promoter was used.

### Characterization of Kinetic Parameters of *Zr*Fsy1

The substrate specificity of *Zr*Fsy1 estimated by the growth experiments (Figures 2 and 3) was verified by measuring the initial rate of uptake of hexoses either directly (fructose) or as the ability of another putative substrate to competitively inhibit the uptake of fructose in *hxt*-null *S. cerevisiae* cells expressing *ZrFSY1* from its own promoter. A detailed kinetic characterization of *Zr*Fsy1 using [U-^14^C] fructose revealed that *Zr*Fsy1 is a high-affinity low-capacity fructose transporter system, with K_m_ 0.45±0.07 mM and V_max_ 0.57±0.02 mmol h^−1^ (gdw) ^−1^, and that the transport of radioactive fructose by *Zr*Fsy1 was inhibited by sorbose but not by glucose or galactose (results not shown).

The fructose transport capacity (V_max_) of cells expressing *ZrFSY1* under the *ScNHA1* promoter was about 70% lower than that of cells expressing *ZrFSY1* under its own promoter (results not shown), which explains the slower growth of the former in low-fructose media (Figure 3).

**Figure 4:**
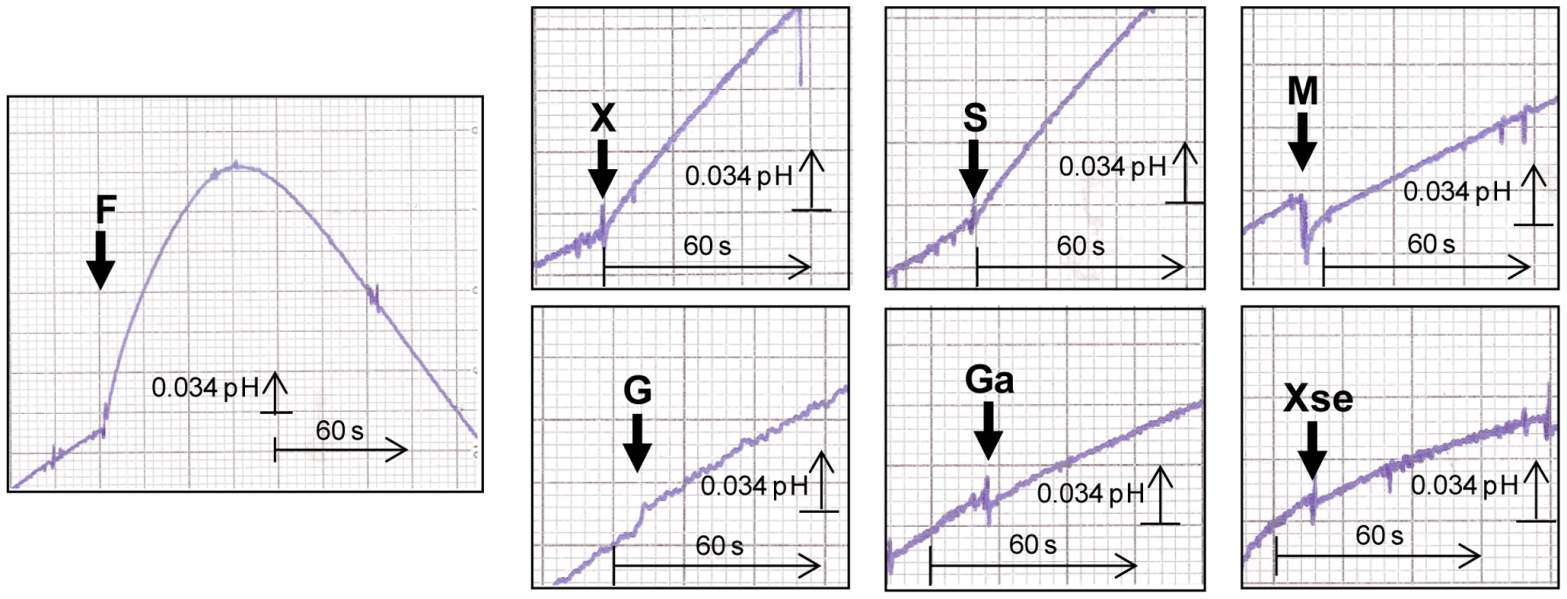
Symport activity in *S. cerevisiae* expressing *Zr*Fsy1. Changes in extracellular pH upon addition of 10 mM (final concentration) fructose (F), xylitol (X), sorbose (S), mannitol (M), glucose (G), galactose (Ga) or xylose (Xse) to aqueous cell suspension of *S. cerevisiae* cells grown in YNB medium with 2% fructose and expressing *ZrFSY1* from its own promoter. The arrows indicate the times of sugar/polyol addition. Data are representative of at least two independent experiments.

To determine the mechanism of sugar transport via *Zr*Fsy1, *i.e.* whether it is a passive facilitated diffusion or an active symport with protons, we monitored the changes in extracellular pH of an unbuffered cell suspension upon the addition of a fructose pulse. As a fast alkalinisation of the external medium was observed upon fructose addition (followed by an acidification resulting from the activation of the plasma-membrane Pma1 H^+^-ATPase activity, Figure 4) we assumed that *Zr*Fsy1 is a fructose/H^+^ symporter, since the extracellular alkalinisation is due to a reduction in the extracellular H^+^ concentration as protons are entering the cells by symport with added fructose. This increased extracellular alkalinisation effect, though much weaker, was also observed upon the addition of xylitol and sorbose, indicating that *Zr*Fsy1 also transports these substrates, but at a much lower rate than fructose. No increased alkalinisation of the cell suspension was observed after glucose, galactose, mannitol or xylose addition (the slope of the baseline before adding sugars/polyols remained constant after the addition of these putative substrates, Figure 4). No increased alkalinisation was observed in control cells that did not express *Zr*Fsy1 (results not shown).

The kinetic parameters K_m_ 0.38±0.05 mM and V_max_ 0.61±0.23 mmol h^−1^ (gdw) ^−1^, determined by symport assays with fructose concentrations in the range 0.1 mM to 40 mM, were similar to those obtained from radiolabelled fructose assays, pointing to a fructose:H^+^ stoichiometry of 1∶1.

### Expression and Activity of *Zr*Fsy1 in *Z. rouxii* CBS 732

**Figure 5:**
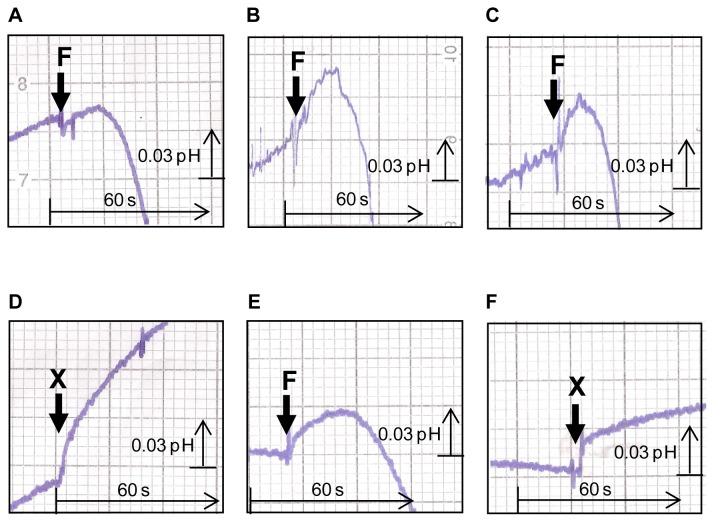
Symport activity in *Z. rouxii* CBS 732. Effect on extracellular pH elicited by addition of fructose (F) or xylitol (X) to final concentration of 10 mM to unbuffered aqueous cell suspensions of *Z. rouxii* CBS 732 grown in YNB medium with 0.5% fructose (A), 0.05% fructose (B), 0.1% fructose (C and D), 2% mannitol (E and F). The arrows indicate the times of fructose/xylitol addition. Data are representative of at least two independent experiments.

Surprisingly, when we monitored the changes in extracellular pH with *Z. rouxii* cells grown in low fructose (0.5%), no clear alkalinisation was observed after the addition of a fructose pulse, merely a rapid acidification was observed, probably resulting from the activation of Pma1 H^+^-ATPase (Figure 5 A). This result suggested that in *Z. rouxii* cells grown in 0.5% fructose, the *Zr*Fsy1 transporter is either not expressed enough or is not functional.

**Figure 6:**
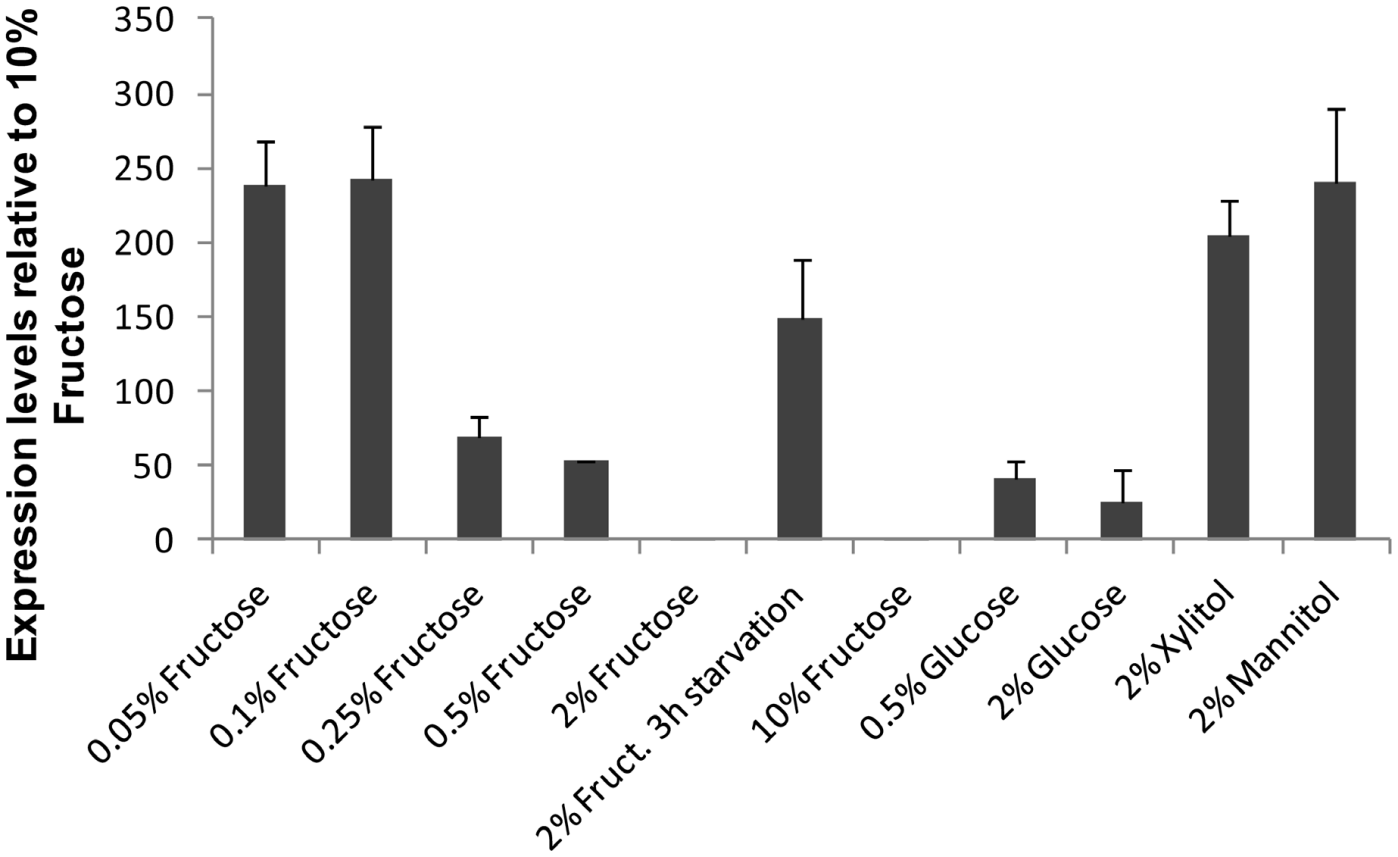
*ZrFSY1* expression in *Z. rouxii* CBS 732. Reverse transcription quantitative-PCR (RT-qPCR) analysis of *ZrFSY1 g*ene expression in *Z. rouxii* CBS 732 cells grown in YNB medium containing various carbon sources at 30°C. All media were inoculated (initial OD_640_ 0.02–0.06) with cells pre-grown on 2% glucose. Exponentially growing cells were collected at OD_640_ 0.6–1.2 and RNA was extracted and analyzed by RT-qPCR. The relative expression of *ZrFSY1* was normalized against *ACT1* expression and the expression levels are represented as relative to the lowest value (10% fructose). The results are expressed as mean ± S.D. from at least two independent experiments.

To ascertain under which growth conditions the fructose/H^+^ symporter *ZrFSY1* is expressed we analysed the level of expression of this gene in *Z. rouxii* cells grown on various carbon sources. The results obtained (Figure 6) showed that *ZrFSY1* is only highly expressed in *Z. rouxii* cells cultivated in media with extremely low fructose concentrations, *e.g.* 0.05% and 0.1%, and in media with non-fermentable carbon sources, such as mannitol and xylitol. Consequently, the alkalinisation of external media upon the addition of a fructose or xylitol pulse was only observed in cells grown in conditions where a higher level of expression was observed, *i.e.* at extremely low concentrations of fructose (Figure 5 B, C and D and Figure 6). Also in cells grown in the presence of 2% mannitol, the alkalinisation was observed upon the addition of fructose or xylitol (Figure 5 E, F). It is worth noting that the transport of xylitol via *Zr*Fsy1 did not activate the Pma1 H^+^-ATPase, as no acidification was observed after the alkalinisation (Figure 5 D, F).

Higher concentrations of fructose repressed the expression of *ZrFSY1* (Figure 6) and a subsequent starvation led to an increase in expression observable within 3 hours. However, after three hours of starvation, the addition of a fructose pulse to cells did not result in a measurable extracellular alkalinisation (results not shown), indicating that though the *ZrFSY1* mRNA is synthesized within three hours of starvation (Figure 6), functional transporters are not present in the plasma membrane.

**Figure 7:**
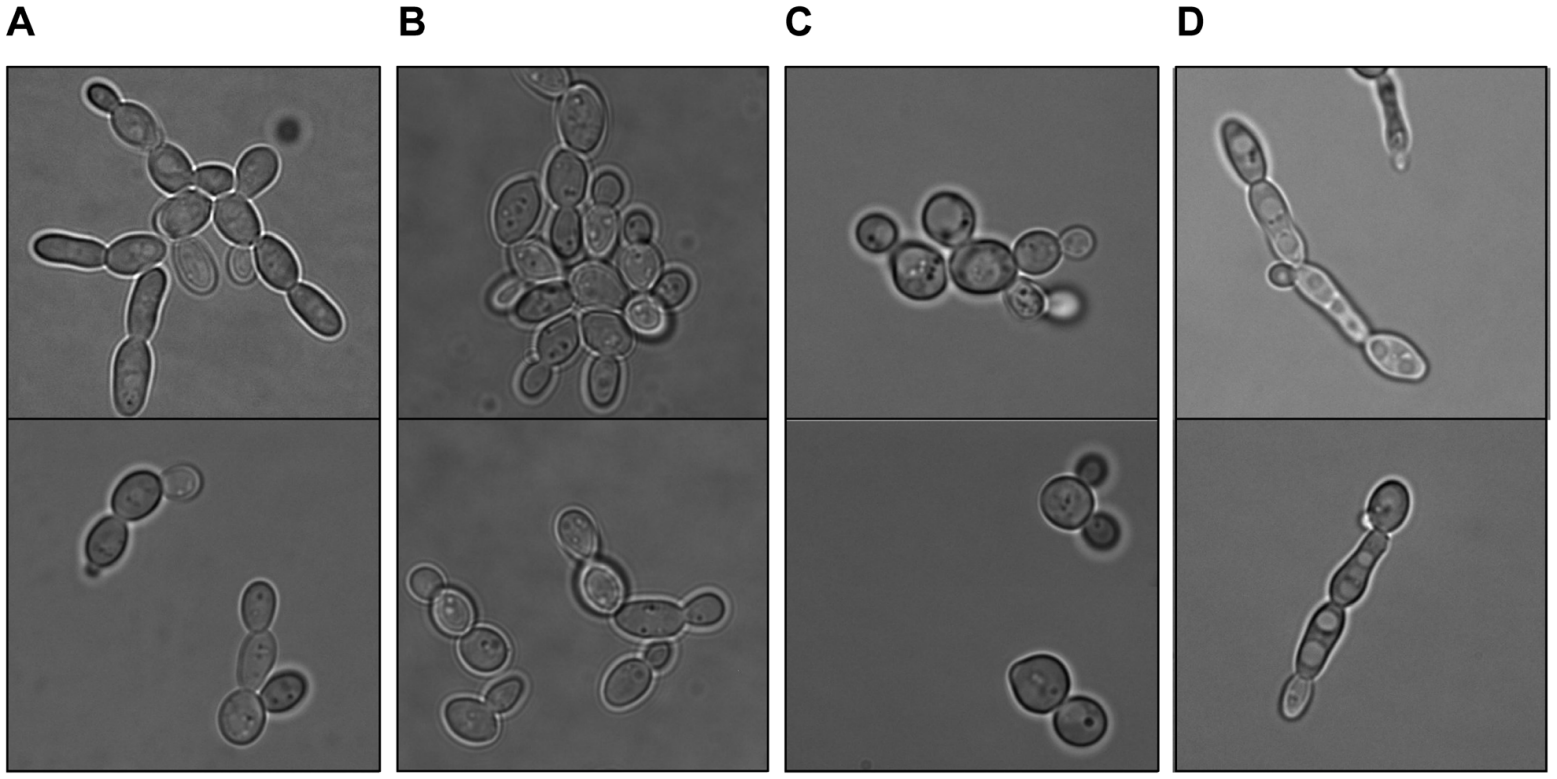
*Z. rouxii* CBS 732 morphology with various carbon sources. Phase contrast images of exponentially grown (OD_640_ ≈ 1) cells of *Z. rouxii* CBS 732 grown on YNB medium with 2% fructose (A), 2% glucose (B), 2% xylitol (C) and 2% mannitol (D).

When *Z. rouxii* cells pre-grown on YBN media with 2% glucose were transferred (batch assays in Erlenmeyer flasks with a volume ratio medium/Erlenmeyer capacity of 1∶3.3) to media with 2% mannitol or 2% xylitol, they behaved completely differently than when transferred to fructose or glucose media, having a much longer lag phase (about 40 h), much lower specific growth rates (0.059±0.009 h^−1^ for 2% mannitol; 0.013±0.001 h^−1^ for 2% xylitol; *versus* 0.150±0.015 h^−1^ for 2% fructose), and lower final biomass. This probably reflected the poor ability of *Z. rouxii* to utilise these non fermentable carbon sources. Furthermore, *Z. rouxii* cells in xylitol medium became more spherical, whereas cells cultivated in mannitol became more elongated, forming pseudohyphae (Figure 7), especially in the stationary phase of growth.

### Phenotypes of *ZrFSY1* Deletion

**Figure 8:**
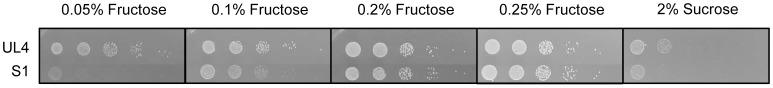
Effect of *ZrFSY1* deletion on *Z. rouxii* growth on solid media. Growth of *Z. rouxii* UL4 (wt) and deletion mutant S1 (*Zrfsy1Δ*) cells grown in YNB medium with various amounts of fructose after 3 days (fructose) or 8 days (sucrose) of incubation at 30°C. Results from one of two independent experiments are shown.

**Figure 9:**
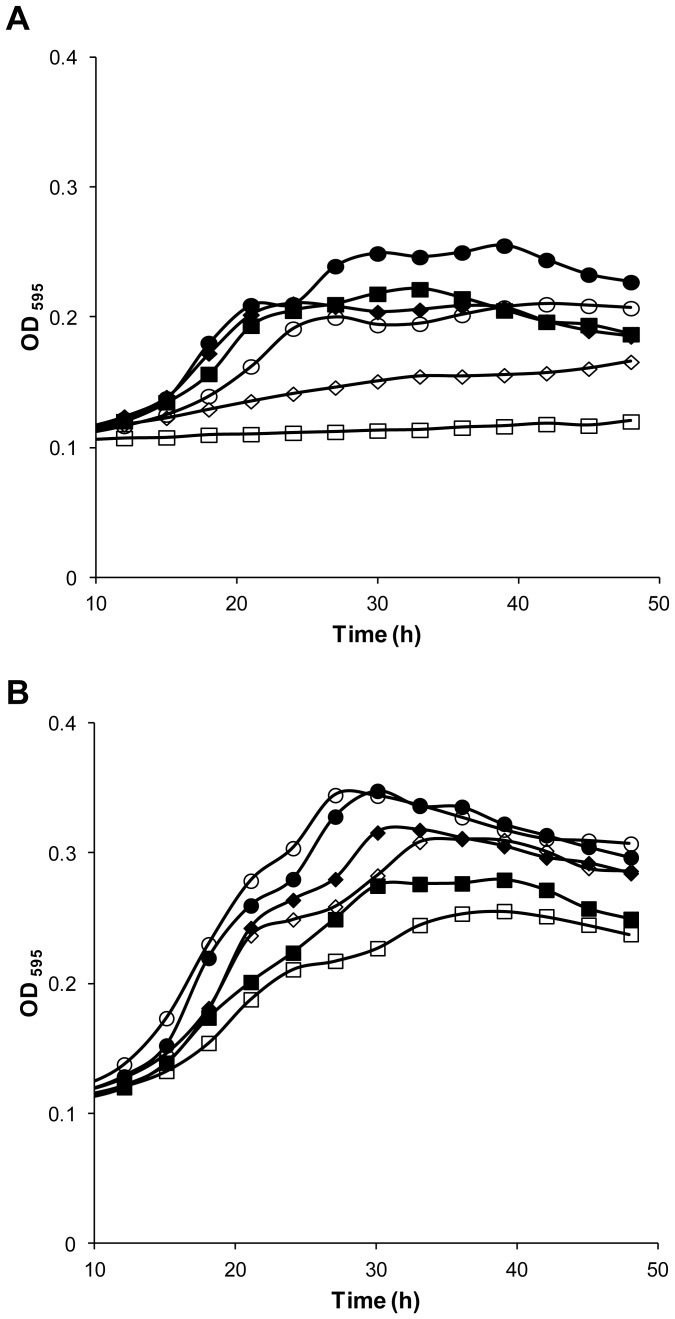
Effect of *ZrFSY1* deletion on *Z. rouxii* growth on liquid media. Growth curves of *Z. rouxii* UL4 (wt; filled symbols) and deletion mutant S1 (*Zrfsy1Δ*; empty symbols) cells grown in YNB medium supplemented with various concentrations of fructose: (A): 0.05% (▪,□), 0.1% (♦,◊), 0.15% (•,○); (B): 0.2% (▪,□), 0.25% (♦,◊), 0.5% (•,○). Data are representative of two independent experiments.

To test the role of *Zr*Fsy1 in *Z. rouxii* cells, the corresponding gene was deleted and growth phenotypes of the resulting mutant were compared with those of the parental strain. A clear growth phenotype was only observed if the cells were grown in fructose concentrations lower than 0.2%, *i.e.* 11 mM. Under these conditions, the absence of *ZrFSY1* reduced the growth rate (Figures 8, 9), especially at extremely low (0.05%) concentrations, where the deletion of the symporter almost abolished growth. The observed phenotypes correlated well with Real-time PCR data and indicated that the *Zr*Fsy1 symporter is the main fructose transporter in *Z. rouxii* growing at those very low fructose concentrations. Slower growth of the S1 mutant strain was also observed when sucrose was used as the sole carbon source (Figure 8).

## Discussion

Sugar/H^+^ symporters generally operate at very low external sugar concentrations, where facilitated diffusion would not be efficient enough. Since *Z. rouxiii* is generally isolated from high sugar environments [1] where sugars enter the cells via low-affinity facilitators (as is the case for fructose and *Zr*Ffz1 and *ZrF*fz2) without the need to spend energy on symport with protons, and since the initial assessment of hexose symporter activity in *Z. rouxiii* was negative (S. Sousa-Dias and M. C. Loureiro-Dias, unpublished data) the existence of a putative sugar-proton symporter in the *Z. rouxiii* genome was surprising.

Phylogenetically, *Zr*Fsy1 belongs to the Sugar Porter family (Figure S2), as do most of the sugar transporters characterized so far, being therefore unrelated to the other *Z. rouxii* fructose facilitators *Zr*Ffz1 and *Zr*Ffz2 recently characterized, which have low protein sequence similarity to the members of the Sugar Porter family and seem to form a new family of hexose transporters [14]. The symporter *Zr*Fsy1 is closely related to the other already characterized specific fructose/H^+^ symporters, although with a lower fructose transport capacity than that of *S. pastorianus* Fsy1 [6], but similar to that of *S. cerevisiae* Fsy1 EC1118, identified in a wine strain [13] (Table 1).

**Table 1:** Kinetic parameters of Fsy1-type transporters and Ffz facilitators for fructose transport.

Protein	K_m_ (mM)	V_max_ (mmol h^−1^ (gdw) ^−1^)	Reference
*Sp*Fsy1	0.16±0.02	3.8±0.2	[6]
*Kl*Frt1	0.16±0.02	-	[9]
*Bc*Frt1	1.1	-	[10]
Fsy1 EC1118	0.24±0.04	0.93±0.08	[13]
*Zr*Fsy1	0.45±0.07	0.57±0.02	This study
*Zr*Ffz1	424.2±163.1	12.7±3.3	[14]
*Zr*Ffz2	204.7±51.1	4.51±0.56	[14]
*Zb*Ffz1	80.4	3.3	[15]

In *Z. rouxii*, fructose/H^+^ symport activity was not detected when the cells were cultivated in 0.5% fructose, which had been previously observed to be an induction condition for the expression of the Fsy1 symporter in *S. pastorianus* and *S. bayanus* [7]. This difference suggests that *Zr*Fsy1 is even more tightly regulated by external fructose concentration, being only derepressed at extremely low fructose concentrations (lower than 0.2%) or in the presence of non-fermentable carbon sources. Expression of the Fsy1 symporter in *S. pastorianus* and *S. bayanus* was also detected with the non-fermentable carbon source ethanol, though the level of expression was lower than with 0.5% fructose. In these yeasts, *FSY1* is repressed in the presence of higher concentrations of both fructose and glucose, however, glucose is probably a stronger repressor than fructose [7]. Our results show that in *Z. rouxii,* fructose has a stronger repressing effect on *ZrFSY1* than glucose (observed with 2% sugars, Figure 6), avoiding a waste of proton motive force through the symporter when fructose concentration is high.

As an uptake of sugar by a symport mechanism is costly in terms of cellular energy, when fructose is abundant (and this is the case in the majority of environments from which *Z. rouxii* has been isolated, *e.g.* concentrated black-grape must from which the CBS 732 strain was isolated [31]), it is preferably transported via transporters utilising a facilitated diffusion mechanism, Z*r*Ffz1 and *Zr*Ffz2 [14]. Our results show that under these conditions, the *Zr*Fsy1 symporter is not present in *Z. rouxii* cells. Its expression is only derepressed at very low fructose concentrations, when transport via facilitators is not efficient enough due to their low affinity. At low fructose concentrations, *Zr*Fsy1 becomes the main transporter ensuring the uptake of the required carbon source, as confirmed by the phenotypes of the *Zrfsy1Δ* mutant strain. The role of *Zr*Fsy1 in providing the carbon source for cell growth was also observed with sucrose, although growth was very slow. *Z. rouxii* cells grow on sucrose medium more slowly than on glucose or fructose media, probably due to a low efficiency of their invertase. The growth of *Z. rouxii* CBS 732 on sucrose is described as weak, deficient or negative in the CBS database (http://www.cbs.knaw.nl/). As for sucrose fermentation, there are variations among the *Z. rouxii* strains. Most *Z. rouxii* strains isolated from vegetable extracts with high sugars are unable to ferment sucrose, only two of them ferment sucrose and those two are unable to ferment maltose [32]. Even in *Z. rouxii* strains that can ferment sucrose, sucrose fermentation is delayed compared to glucose or fructose fermentation, and their invertase seems to be cryptic in a 3-day-culture but is more expressed upon aging [33].

*Z. rouxii* CBS 732 has a notable preference for fructose and glucose as carbon sources, as it is unable to grow on the majority of other carbon sources that are common for other yeasts, such as maltose or glycerol. Therefore, to test the effect of non-fermentable carbon sources on the expression of *ZrFSY1*, we had to cultivate the cells in xylitol or mannitol, though there is a strong “dislike” effect for those carbon sources that is reflected in the prolonged lag phase, longer duplication times (as high as 53 h for 2% xylitol medium), lower final biomass, and change in the morphology of *Z. rouxii* cells (Figure 7). This morphology effect of mannitol and xylitol is not observed in other yeasts, *e.g.* in the halotolerant yeast *Debaryomyces hanseniii* (results not shown). Pseudohyphae formation in *Z. rouxii* is affected by growth conditions, as cells grown in 2% mannitol medium (having pseudohyphae) revert to their “normal” morphology when transferred to fructose or glucose media, or become more spherical when transferred to xylitol medium (results not shown).

Our results showed that the fructose/H^+^ symporter from *Z. rouxii* is tightly regulated by the amount of fructose in the environment. This also seems to be the case when it is expressed in a *hxt*-null *S. cerevisiae* strain, since very poor growth at high fructose concentrations was observed, even when a constitutive promoter was used, indicating that a post-transcriptional regulation mechanism of *ZrFSY1* in *S. cerevisiae* may be involved. In *Z. rouxii* CBS 732, it is active when the concentration of fructose drops to levels so reduced that the low-affinity fructose facilitators are not able to ensure sufficient transport of this sugar. We believe that the characterization of this symporter could also contribute towards improving the fructose fermentation of *S. cerevisiae*. The heterologous expression of *Zr*Fsy1 in industrial *S. cerevisiae* strains and its high affinity for fructose could be useful for scavenging residual fructose, therefore preventing an unacceptable sweet taste of the fermentation products, and finally, reducing the risk of microbial deterioration in wines.

## Supporting Information

Figure S1 **Representative alignment of Fsy1-like proteins.** Analysis was performed using the MUSCLE web server (Edgar, 2004) for multiple alignments. Conserved regions in the Sugar Porter family are indicated. Represented proteins (and corresponding accession numbers) are: *Kl*Frt1-*K. lactis* fructose symporter (CAC79614.1); *Sb*Fsy1-*S. bayanus* fructose symporter (CCI61478.1); *Sc*EC1118Fsy1-*S. cerevisiae* EC1118 fructose symporter (CAY86682.1); *Se*Fsy1-*S. eubayanus* fructose symporter (CCI61473.1); *Spa*Fsy1-*S. pastorianus* fructose symporter (CAC08232.1); *Su*Fsy1-*S. uvarum* fructose symporter (CCI61480.1); *Zr*Fsy1-*Z. rouxii* fructose symporter (CAR26745.1).
(PPTX)

Figure S2 **Dendrogram based on primary protein sequence homology using the neighbor-joining method (applied to 1000 bootstrap data sets), depicting the phylogenetic relationship between the** ***Zr*** **Fsy1 protein, other members of the Sugar Porter family and the phylogenetically distant Ffz hexose transporters.** Represented proteins (and corresponding accession numbers) are: *Bc*Frt1-*Botrytis cinerea* fructose/H^+^ symporter (AAU87358.1); *Gz*HP-*Gibberella zeae* PH-1 hypothetical protein FG09335.1 (XP_389511.1); *Kl*Frt1-*K. lactis* fructose symporter (CAC79614.1); *Kl*Rag1, *K. lactis* low affinity hexose transporter (XP_453656.1); *Pn*HP-*Phaeosphaeria nodorum* SN15 hypothetical protein SNOG_04266 (XP_001794684.1); *Pt*FruF-*Pyrenophora tritici-repentis* Pt-1C-BFP fructose facilitator (putative) (XP_001935732.1); *Sb*Fsy1-*S. bayanus* fructose symporter (CCI61478.1); *Sc*EC1118Fsy1-*S. cerevisiae* EC1118 fructose symporter (CAY86682.1); *Sc*Hxt1-*S. cerevisiae* hexose transporter 1 (AAB68933.1); *Sc*Hxt2-*S. cerevisiae* hexose transporter 2 (AAA34701.1); *Sc*Hxt3-*S. cerevisiae* hexose transporter 1 (DAA12185.1); *Sc*Hxt4-*S. cerevisiae* hexose transporter 1 (DAA06788.2); *Sc*Hxt5-*S. cerevisiae* hexose transporter 1 (DAA06790.1); *Sc*Hxt7-*S. cerevisiae* hexose transporter 7 (AAB64778.1); *Sc*Hxt14-*S. cerevisiae* hexose transporter 1 (DAA10243.1); *Se*Fsy1-*S. eubayanus* fructose symporter (CCI61473.1); *Spa*Fsy1-*S. pastorianus* fructose symporter (CAC08232.1); *Su*Fsy1-*S. uvarum* fructose symporter (CCI61480.1); *Zb*Ffz1-*Z. bailii* fructose transporter (CAD56485.1); *Zr*Ffz1-*Z. rouxii* fructose transporter (CAR31108.1); *Zr*Ffz2-*Z. rouxii* fructose and glucose transporter (CAR28354.1); *Zr*Fsy1-*Z. rouxii* fructose symporter (CAR26745.1).
(DOCX)

Table S1 **Oligonucleotides used in this study.**
(DOCX)
