# Supplementary material for: ZrFsy1, a High-Affinity Fructose/H+ Symporter from Fructophilic Yeast Zygosaccharomyces rouxii
Source: PLoS One. 2013 Jul 2;8(7):e68165. doi: 10.1371/journal.pone.0068165 (PMC3699521; doi:10.1371/journal.pone.0068165)
Supplement: Figure S2 — Dendrogram based on primary protein sequence homology using the neighbor-joining method (applied to 1000 bootstrap data sets), depicting the phylogenetic relationship between the Zr Fsy1 protein, other members of the Sugar Porter family and the phylogenetically distant Ffz hexose transporters. Represented proteins (and corresponding accession numbers) are: BcFrt1-Botrytis cinerea fructose/H+ symporter (AAU87358.1); GzHP-Gibberella zeae PH-1 hypothetical protein FG09335.1 (XP_389511.1); KlFrt1-K. lactis fructose symporter (CAC79614.1); KlRag1, K. lactis low affinity hexose transporter (XP_453656.1); PnHP-Phaeosphaeria nodorum SN15 hypothetical protein SNOG_04266 (XP_001794684.1); PtFruF-Pyrenophora tritici-repentis Pt-1C-BFP fructose facilitator (putative) (XP_001935732.1); SbFsy1-S. bayanus fructose symporter (CCI61478.1); ScEC1118Fsy1-S. cerevisiae EC1118 fructose symporter (CAY86682.1); ScHxt1-S. cerevisiae hexose transporter 1 (AAB68933.1); ScHxt2-S. cerevisiae hexose transporter 2 (AAA34701.1); ScHxt3-S. cerevisiae hexose transporter 1 (DAA12185.1); ScHxt4-S. cerevisiae hexose transporter 1 (DAA06788.2); ScHxt5-S. cerevisiae hexose transporter 1 (DAA06790.1); ScHxt7-S. cerevisiae hexose transporter 7 (AAB64778.1); ScHxt14-S. cerevisiae hexose transporter 1 (DAA10243.1); SeFsy1-S. eubayanus fructose symporter (CCI61473.1); SpaFsy1-S. pastorianus fructose symporter (CAC08232.1); SuFsy1-S. uvarum fructose symporter (CCI61480.1); ZbFfz1-Z. bailii fructose transporter (CAD56485.1); ZrFfz1-Z. rouxii fructose transporter (CAR31108.1); ZrFfz2-Z. rouxii fructose and glucose transporter (CAR28354.1); ZrFsy1-Z. rouxii fructose symporter (CAR26745.1). (DOCX) [file pone.0068165.s002.docx]

**Figure S2. Dendrogram based on primary protein sequence homology using the neighbor-joining method (applied to 1000 bootstrap data sets), depicting the phylogenetic relationship between the *Zr*Fsy1 protein, other members of the Sugar Porter family and the phylogenetically distant Ffz hexose transporters.** Represented proteins (and corresponding accession numbers) are: *Bc*Frt1-*Botrytis* *cinerea* fructose/H^+^ symporter (AAU87358.1); *Gz*HP-*Gibberella zeae* PH-1 hypothetical protein FG09335.1 (XP_389511.1); *Kl*Frt1-*K. lactis* fructose symporter (CAC79614.1); *Kl*Rag1, *K. lactis* low affinity hexose transporter (XP_453656.1); *Pn*HP-*Phaeosphaeria nodorum* SN15 hypothetical protein SNOG_04266 (XP_001794684.1); *Pt*FruF-*Pyrenophora tritici-repentis* Pt-1C-BFP fructose facilitator (putative) (XP_001935732.1); *Sb*Fsy1-*S. bayanus* fructose symporter (CCI61478.1); *Sc*EC1118Fsy1-*S. cerevisiae* EC1118 fructose symporter (CAY86682.1); *Sc*Hxt1-*S. cerevisiae* hexose transporter 1 (AAB68933.1); *Sc*Hxt2-*S. cerevisiae* hexose transporter 2 (AAA34701.1); *Sc*Hxt3-*S. cerevisiae* hexose transporter 1 (DAA12185.1); *Sc*Hxt4-*S. cerevisiae* hexose transporter 1 (DAA06788.2); *Sc*Hxt5-*S. cerevisiae* hexose transporter 1 (DAA06790.1); *Sc*Hxt7-*S. cerevisiae* hexose transporter 7 (AAB64778.1); *Sc*Hxt14-*S. cerevisiae* hexose transporter 1 (DAA10243.1); *Se*Fsy1-*S. eubayanus* fructose symporter (CCI61473.1); *Spa*Fsy1-*S. pastorianus* fructose symporter (CAC08232.1); *Su*Fsy1-*S. uvarum* fructose symporter (CCI61480.1); *Zb*Ffz1-*Z. bailii* fructose transporter (CAD56485.1); *Zr*Ffz1-*Z. rouxii* fructose transporter (CAR31108.1); *Zr*Ffz2-*Z. rouxii* fructose and glucose transporter (CAR28354.1); *Zr*Fsy1-*Z. rouxii* fructose symporter (CAR26745.1).

**Ffz family**

**Sugar Porter family**

Ffz-like transporters

Hxt-like transporters

Fsy1-like transporters
