# Supplementary material for: ZrFsy1, a High-Affinity Fructose/H+ Symporter from Fructophilic Yeast Zygosaccharomyces rouxii
Source: PLoS One. 2013 Jul 2;8(7):e68165. doi: 10.1371/journal.pone.0068165 (PMC3699521; doi:10.1371/journal.pone.0068165)
Supplement: Table S1 — Oligonucleotides used in this study. (DOCX) [file pone.0068165.s003.docx]

**Table S1. Oligonucleotides used in this study.**

| **Primer** | **Sequence (5´-3´)*** |
| --- | --- |
|  |  |
| ZrFSY1-N-F | 5’−TGTACATTATAAAAAAAAATCCTGAACTTAGCTAGATATTATGAAGTTTTCTACTTGGCGG−3’ |
| ZrFSY1-R | 5’−CACCTTTAGACATTTTAATAAAGCTCCGGAGCTTGCATGCATAGCTTAGTTTACCTCTTTTCAACCA−3’ |
| ZrFSY1-S-F | 5’−CCGATTCATTCCCGAATTCGAGCTCGGTACCCGGGGATCCTATCCATATCAATTAAGCCTG−3’ |
| ZrFSY1-P1 | 5’−TTCATTGAGCACTAGATCTGC−3’ |
| ZrFSY1-P2 | 5’−TACCGATAACCACTGCAATACC−3’ |
| ZrACT1-P1 | 5’−CCGCTTTGGCTCCATCTTCTAT−3’ |
| ZrACT1-P2 | 5’−GTCCGCTTTCGTCGTATTCTTG−3’ |
| ZrFSY1-Kan-F | 5’−CCGGTAAGTTCACCCATCCATATTTTTCAAGCTTCTCTAACAGGATTTTTCAGATACGTAGAAGAAGTTGTGTCAAAGAATAACAGCGGTTTCGTACGCTGCAGGTCGAC−3’ |
| ZrFSY1-Kan-R | 5’−TGAGAACCTGTAAAGAATCAACTGTTTTTGACTCTCTTCGCTCCCCTATCCATTCTGTCTCTAATAGCTTAGTTTACCTCTTTTCAACCAGCATAGGCCACTAGTGGATCTG−3’ |
| KANX-R1 | 5’−CTCTGGCGCATCGGGC−3’ |
| KANX-F1 | 5’−CATTTGATGCTCGATGA−3’ |
| ZrFSY1-368-upF | 5’−AGGATGATCACTGACTACTTGC−3’ |
| ZrFSY1-246d-R | 5’−GCATCATGTAATACGACGACC−3’ |

*Sequences homologous to the *Z. rouxii* *FSY1* gene are underlined in the primers used for cloning or deletion via homologous recombination.
